# A five-country study of front- and back-of-package nutrition label awareness and use: patterns and correlates from the 2018 International Food Policy Study

**DOI:** 10.1017/S1368980022002257

**Published:** 2022-10-26

**Authors:** Jasmin Bhawra, Sharon I Kirkpatrick, Marissa G Hall, Lana Vanderlee, James F Thrasher, Alejandra Jáuregui de la Mota, David Hammond

**Affiliations:** 1 School of Occupational and Public Health, Faculty of Community Services, Toronto Metropolitan University, Toronto, ON, Canada; 2 School of Public Health Sciences, Faculty of Health, University of Waterloo, Waterloo, ON N2L 3G1, Canada; 3 Department of Health Behavior, Gillings School of Global Public Health, and Lineberger Comprehensive Cancer Center, University of North Carolina, Chapel Hill, NC, USA; 4 École de Nutrition, Centre Nutrition, Santé et Société (Centre NUTRISS) and Institut Sur la Nutrition et les Aliments Fonctionnels (INAF), Université Laval, Québec, Canada; 5 Department of Health Promotion, Education and Behavior, Arnold School of Public Health, University of South Carolina, Columbia, SC, USA; 6 Department of Physical Activity and Healthy Lifestyles, National Institute of Public Health, Cuernavaca, Mexico

**Keywords:** Nutrition labelling, Food policy, Label awareness, Label use, International

## Abstract

**Objective:**

This study aimed to identify correlates of nutrition label awareness and use, particularly subgroup differences among consumers. Two label types were assessed: (1) nutrition facts tables (NFt) in Australia, Canada, Mexico, UK, and USA and (2) front-of-package (FOP) labels, including mandatory Guideline Daily Amounts (Mexico), voluntary Health Star Ratings (Australia) and voluntary Traffic Lights (UK).

**Design:**

Respondents were recruited using Nielsen Consumer Insights Global Panel (*n* 21 586) and completed online surveys in November–December 2018. Linear regression and generalised linear mixed models examined differences in label use and awareness between countries and label type based on sociodemographic, knowledge-related and dietary characteristics.

**Setting:**

Australia, Canada, Mexico, UK and USA.

**Participants:**

Adults (≥18 years).

**Results:**

Respondents from the USA, Canada and Australia reported significantly higher NFt use and awareness than those in Mexico and the UK. Mexican respondents reported the highest level of FOP label awareness, whereas UK respondents reported the highest FOP label use. NFt use was higher among females, ‘minority’ ethnic groups, those with higher nutrition knowledge and respondents with ‘adequate literacy’ compared with those with ‘high likelihood of limited literacy’. FOP label use was higher among those with a ‘high likelihood of limited literacy’ compared with ‘adequate literacy’ across countries.

**Conclusions:**

Lower use of mandatory Guideline Daily Amount labels compared with voluntary FOP labelling systems provides support for Mexico’s decision to switch to mandatory ‘high-in’ warning symbols. The patterns of consumer label use and awareness across sociodemographic and knowledge-related characteristics suggest that simple FOP labels may encourage broader use across countries.

Non-communicable diseases including cardiovascular disease, type 2 diabetes and obesity are the world’s leading causes of premature death and disability, with dietary intake being an important risk factor^(1)^. In recent decades, a global dietary shift towards highly processed foods – including ultra-processed foods – has contributed to poor overall diet quality^(1–3)^. Ultra-processed foods are ‘formulations of food substances often modified by chemical processes and then assembled into ready-to-consume, hyper-palatable food and drink products using flavours, colours, emulsifiers, and a myriad of other cosmetic additives’^(2)^. These foods typically contain high amounts of Na, sugar, saturated or trans fats, leading to energy-dense, nutrient-poor food environments^(2–4)^.

Given that ultra-processed foods constitute more than half of energy intake in high-income countries including Canada, the USA and the UK^(2–5)^, and between one-fifth to one-third of energy intake in middle-income countries such as Mexico and Brazil^(2,6,7)^, governments have adopted policy measures, such as nutrition labelling, to support healthy eating^(8)^. Nutrition labels are found on packaged foods and provide consumers with nutrient information at the point-of-purchase to aid informed decision-making in an increasingly processed food landscape^(3,8)^, while also incentivising the food industry to reformulate towards healthier nutritional profiles^(1,8,9)^. Nutrition labels implemented to date include back- or side-of-package nutrition facts tables (NFt) and front-of-package (FOP) labelling systems. NFt feature quantitative information on nutrient amounts, whereas FOP labels focus on simplified, interpretive information, often using symbols instead of numeric information to promote comprehension^(10,11)^.

In most cases, NFt implemented in different countries have a similar appearance and information content^(10)^. In contrast, most FOP labels are voluntary and differ across countries. They may be nutrient-specific or interpretive summary indicator systems^(11)^. Nutrient-specific FOP labelling systems highlight select nutrients of public health concern in the product, such as Mexico’s former Guideline Daily Amount label, which reinforces information also in the NFt, including calories, total sugars, saturated fats and Na^(11)^. Summary indicator systems summarise nutrient content and interpret product healthfulness using algorithms to provide a score or ordinal ranking of the overall product^(12)^. For example, Australia’s Health Star Rating assigns 0·5 to 5 stars to a food product, with higher star ratings corresponding with healthier options^(11)^, whereas the UK has adopted a nutrient-specific Traffic Lights system indicating amounts of total fat, saturated fats, total sugars and Na in a product using colour-coding (high = red, medium = yellow and low = green)^(11)^.

FOP labelling policies may be voluntarily implemented or mandatory in a given jurisdiction. The Mexican Guideline Daily Amount system was initially industry-led and later made mandatory by government, unlike the Health Star Rating and Traffic Light systems, which are government-led and voluntary. Voluntary policies provide food manufacturers with an option to opt out of implementing FOP labels. For example, the Health Star Rating appears on less than one-third of packaged food products^(13)^, whereas in countries such as Chile and Mexico, FOP labels are mandatory and must be displayed on packaged products that exceed nutrient thresholds.

Consumer awareness and use of nutrition labels are key indicators of the visibility and effectiveness of labelling policies and related nutrition education initiatives. Awareness is indicative of consumers’ attention and exposure to labelling policy, thus precedes label use^(14)^. Label understanding is critical to – but does not guarantee – label use^(14)^. Label awareness, understanding and use are influenced by a range of factors, which have largely been explored via experimental or ‘pre-implementation’ studies^(11)^. A growing number of pre-implementation studies suggest FOP labels are easier to understand than NFt, particularly among consumers with lower education and income^(9,15)^. In comparison, greater use of NFt has been observed among women and those with higher income and education^(15,16)^. Moreover, consumers with specific motivation (i.e. diet-or weight-related goals), dietary behaviours (i.e. vegetarianism) and with prior nutrition knowledge have been associated with higher NFt label awareness and use^(12,14,17–19)^. Given the relative dearth of post-implementation research and recency of FOP labelling policies, research is needed to understand whether consumers who use FOP labels are similar to those who use NFt.

There is also little post-implementation data that compare use and awareness of FOP labels across different countries, or NFt to FOP label use within countries with both label types. These evidence gaps limit our ability to evaluate the uptake and effectiveness of different labelling policies across subgroups (i.e. among consumers with higher *v*. lower health literacy status) and countries, which may inform policy adoption or dissemination strategies in countries considering FOP labelling systems. This study thus aimed to examine differences in nutrition label awareness and use across five countries (Australia, Canada, Mexico, the UK and the USA), three of which have government-led FOP labelling policies in place (Australia, Mexico and the UK). In particular, this study explored between-country differences in NFt use and awareness; correlates of NFt and FOP label use and awareness, including sociodemographic, dietary and knowledge-related characteristics; and NFt *v*. FOP label use and awareness in countries with both.

## Methods

### Study design and participants

This study used cross-sectional data from the 2018 wave of the International Food Policy Study^(20)^. Respondents aged 18 years and over and were recruited in Australia, Canada, Mexico, the UK and the USA via Nielsen Consumer Insights Global Panel and their partners’ panels and completed web-based surveys between November and December 2018. The Nielsen panels use probability and non-probability recruitment methods in each country. Email invitations were sent to a random sample of panellists after targeting for age and sex in each country. Quotas were applied to facilitate recruitment of a diverse sample that approximated known proportions in each country for males and females in four age groups: 18–29, 30–44, 45–64, and 65 years and over. Respondents were queried about a range of topics related to nutrition and the food environment, including food purchasing, dietary behaviours, nutrition knowledge and perceptions of national-level food policies. Surveys were conducted in English in Australia and the UK; Spanish in Mexico; English or French in Canada; and English or Spanish in the USA^(20)^.

All respondents provided consent prior to completing the survey and received remuneration in accordance with their panel’s usual incentive structure (e.g. points-based, monetary rewards or chances to win prizes).

Of the 22 824 respondents who completed the 2018 IFPS survey, a subsample of 21 586 respondents from Australia (*n* 3901), Canada (*n* 4107), Mexico (*n* 4012), the UK (*n* 5121) and the USA (*n* 4445) were included in the current study. Those with missing data for ethnicity (*n* 296), income adequacy (*n* 182), education (*n* 69), food shopping role (*n* 29), dietary efforts (*n* 122), health literacy status (*n* 29); self-reported NFt awareness (*n* 157) and use (*n* 184); self-reported FOP label awareness (*n* 201) and use (*n* 201); and Food Processing Knowledge (FoodProK) (*n* 17) were excluded from analyses. Respondents with missing data were not different with respect to label awareness and use compared with the rest of the sample (data not shown). The median time to complete the survey across all countries was 40 min.

### Measures

#### Self-reported awareness and use of food labels

Label awareness was measured by showing respondents country-specific NFt (Table 1) and asking, ‘Have you seen this type of food label on packages or in stores?’ (response options were never/rarely/sometimes/often/all the time). Label use was measured by asking, ‘How often do you use this type of food label when deciding to buy a food product?’ (never/rarely/sometimes/often/all the time). These measures were adapted from the 2014 US Food and Drug Agency Health and Diet Survey^(21)^. After answering questions about the NFt, respondents from Australia, Mexico and the UK were shown images of the FOP labels in place in their countries at the time, including voluntary Health Star Ratings, mandatory Guideline Daily Amounts, and voluntary multiple Traffic Lights, respectively (Table 1), and asked to respond to the same measures of label awareness and use. All labelling variables were queried using a five-point response scale and analysed as continuous variables. Potential correlates of label awareness and use were identified from the literature and included nutrition knowledge, consumer dietary behaviours, BMI and sociodemographic characteristics.

**Table 1:** Food labels by country in the 2018 International Food Policy Study survey

	Australia	Canada	Mexico	UK	USA
NFt	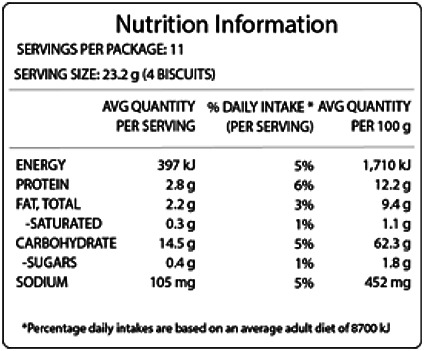	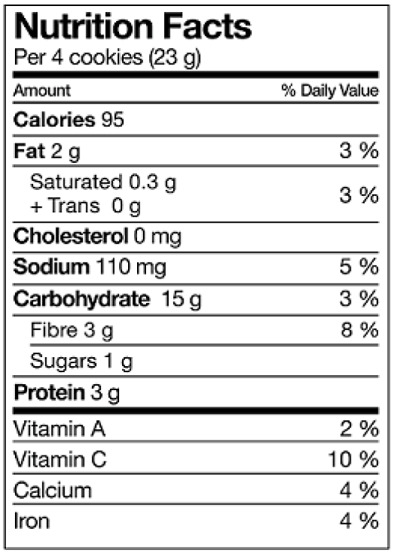	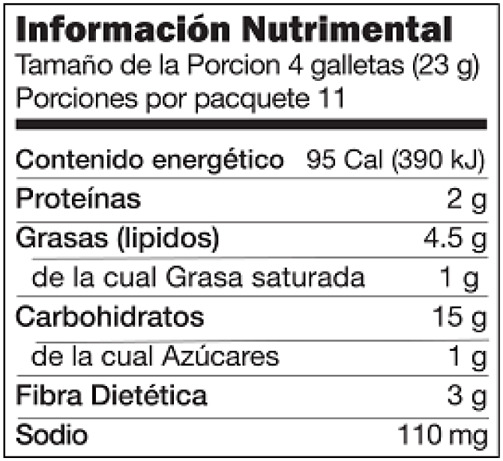	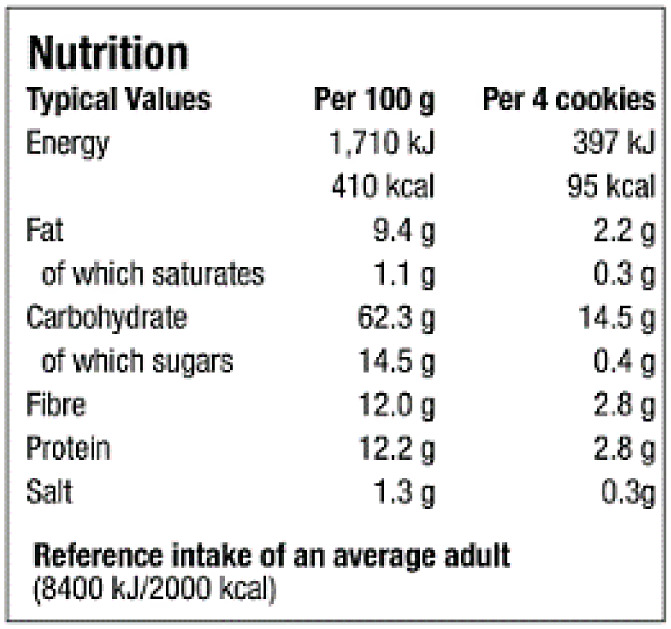	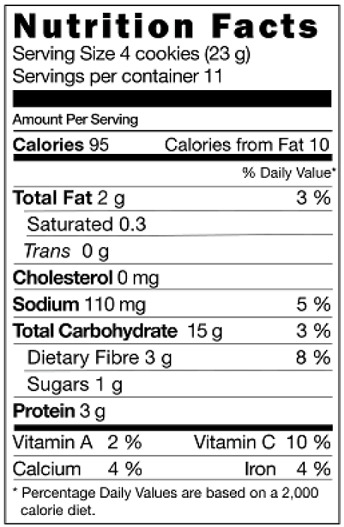
FOP label	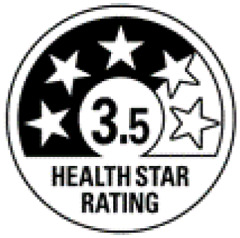 Voluntary Health Star Ratings introduced in 2014	None	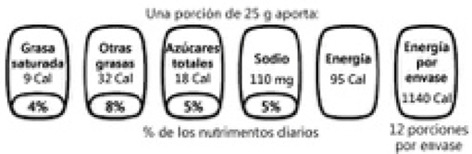 Mandatory Guideline Daily Amounts between 2016 and 2020	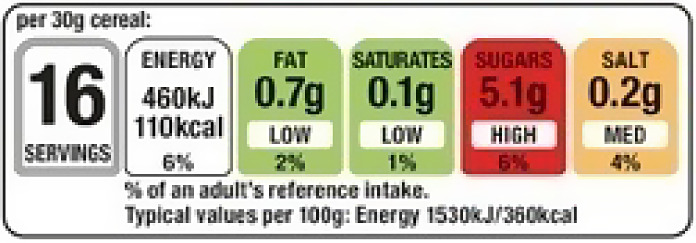 Voluntary Traffic Lights introduced in 2013	None

NFt, nutrition facts table; FOP, front-of-package.

#### Functional nutrition knowledge

Prior nutrition knowledge may influence consumers’ ability and motivation to use nutrition labels^(14,18)^. The survey assessed consumer nutrition knowledge using the FoodProK score, a functional test based on level of processing^(22)^. Respondents viewed and rated images of three food products within four categories: fruits (apple, apple juice and apple sauce), meat (chicken breast, deli chicken slices and chicken nuggets), dairy products (1 per cent milk, cheese block and processed cheese slices) and grains (oats, cereal and cereal bar). Products in each category were selected based on availability in multiple international contexts and to represent different levels of processing according to the NOVA system^(2)^. The twelve product images and corresponding NFt and ingredients lists were displayed one at a time, in random order. For each product, respondents were asked, ‘Overall, how healthy is this food product?’ and answered using a scale of 0 to 10, with 0 representing ‘not healthy at all’ to 10 indicating ‘extremely healthy’.

FoodProK scores were calculated based on the concordance of healthiness ratings within each food category to NOVA classification rankings, with less processed foods representing higher healthiness. Respondents received a full score of 2 if their food product ratings corresponded with the order of NOVA food processing groups (e.g. apple > apple sauce > apple juice). If the respondent ranked 2 of 3 products in a given category in accordance with NOVA (e.g. apple > apple juice > apple sauce), they received a score of 1. Zero was assigned if the respondent’s rankings did not align with those based on NOVA. Scores were summed across the four food categories to create the total FoodProK score (hereon referred to as nutrition knowledge score), ranging from 0 to 8^(23)^.

#### Health literacy status

Respondents completed an adapted version of the Newest Vital Sign in which an ice cream container NFt was shown, and respondents answered six questions that assessed their ability to make mathematical calculations (numeracy), read and apply label information (prose literacy), and understand the label information (document literacy)^(24)^. Based on the number of correct answers, respondents were sorted into one of three literacy categories where a score of 0–1 suggested ‘high likelihood (50 % or more) of limited literacy’; a score of 2–3 indicated ‘possibility of limited literacy’; and a score of 4–6 indicated ‘high likelihood of adequate literacy’^(24)^. The Newest Vital Sign served not only as a proxy measure of health and nutrition literacy, but also as a functional measure of consumer NFt understanding. The NFt images were adapted to include NFt design and layout specific to each country^(20)^. A score between 0 and 6 was calculated based on the number of correct answers, with higher scores corresponding with greater NFt understanding.

#### Consumer dietary behaviours

Diet modification efforts, another possible predictor of label awareness and use^(18,19)^, were measured by asking, ‘Have you made an effort to consume more or less of the following in the past year?’ Respondents answered, ‘consume less’, ‘consume more’ or ‘no effort made’, to a list of nutrients and food categories. This study focused on efforts in five categories that have received increasing attention in policies such as dietary guidelines: ‘trans-fats’, ‘sugar/added sugars’, ‘salt/sodium’, ‘calories’, and ‘processed foods’^(1,2,4)^. A value of -1 was assigned to ‘consume less’, +1 to ‘consume more’, and 0 for ‘no effort made’ for each of the five categories. Five points were added to the sum of the five categories to create a scale ranging from 0 to 10, with 0 representing ‘consume less’ responses to all categories, 10 representing ‘consume more’ responses to all categories and the range between reflecting all other response combinations.

Consumers with specific dietary practices, as well as those with a primary food shopping role in their households, were hypothesised to have greater interest in and exposure to labels^(18,19,25)^. Respondents indicated whether they followed any dietary practices (vegetarian/vegan/pescatarian/a religious practice for eating). Responses were recoded to indicate no specific dietary practices or one or more dietary practice. Food shopping role was captured by asking, ‘Do you do most of the food shopping in your household?’ (Yes/No/Share equally with others)^(20)^.

#### Sociodemographic variables and BMI

To capture differences in nutrition label awareness and use based on sociodemographic characteristics, age group (18–29, 30–44, 45–59 and ≥60 years), sex at birth (female or male), country (Australia, Canada, Mexico, the UK and the USA), and derived variables for education and ethnicity were included in analyses. Given that less than 1 % (*n* 113) of respondents reported a gender different than their biological sex, only the variable ‘sex at birth’ was used in analyses. Education level was categorised in accordance with country-specific criteria, with respondents classified as having ‘low’ (high school completion or lower), ‘medium’ (some post-secondary school qualifications, including some university) or ‘high’ (university degree or higher) levels of education^(20)^. Ethnicity was treated as a binary variable to enable between-country comparisons, with respondents categorised as ‘majority’ in Mexico if they identified themselves as ‘non-Indigenous’, and ‘majority’ in Australia, Canada, the UK and the US if they identified themselves as ‘White’, predominantly English-speaking, or non-Indigenous based on country-specific ethnicity questions^(20)^. Income adequacy, which refers to whether an income is enough to support an individual or household, was assessed by asking, ‘Thinking about your total monthly income, how difficult or easy is it for you to make ends meet?’ (Very difficult/Difficult/Neither easy nor difficult/Easy/Very easy)^(20)^. Income adequacy was used instead of household income to ensure relevance of this measure across countries.

Weight status may play a role in consumers’ use or interest in nutrition labels, particularly among those with weight-related goals^(19)^. Categorisation of BMI followed WHO criteria^(26)^, with self-reported height and weight used to classify respondents based on BMI < 18·5 kg/m2, 18·5 to 24·9 kg/m2, 25·0 to 29·9 kg/m2 and ≥30 kg/m2. Given the large number of cases with missing height and weight data – including those who selected ‘don’t know’ or ‘refuse to answer’ – a separate category for ‘missing’ BMI was created and retained as a response category for analyses.

### Statistical analysis

Descriptive statistics were used to summarise the sample profile and labelling outcomes by country. Three multiple linear regression models were fitted to examine NFt/FOP use and NFt awareness across the five countries. All models were adjusted for sociodemographic characteristics (age, sex, country, income adequacy, education level and ethnicity), consumer dietary behaviours (dietary practices, modification efforts and food shopping role) and BMI. Due to the moderate correlation between the nutrition knowledge score and Newest Vital Sign (*r*_s_ = 0·37, *P* < 0·0001), nutrition knowledge score was added to the main model in a subsequent step to assess the association of nutrition knowledge with the labelling outcomes.

Multiple comparisons were conducted to assess all pairwise contrasts for categorical variables. The Benjamini–Hochberg procedure was applied to decrease the false detection rate following multiple exploratory tests^(27)^. All statistically significant pairwise contrasts were reported after applying the Benjamini–Hochberg procedure, assuming a false discovery rate of 10 %. The models tested two-way interactions between country and the covariates age, sex, ethnicity, education, income adequacy, BMI, health literacy status, dietary practices, dietary efforts, and food shopping role, as research has shown differences in label awareness and use based on these characteristics^(14,15)^.

Generalised linear mixed models were run separately for Australia, the UK and Mexico to test awareness of NFt *v*. FOP labels, and use of NFt *v*. FOP labels. A repeated-measures analysis was used to account for the correlated data within individuals for these measures. Each model included two-way interactions for the individual-level variables above to assess whether awareness/use differed for NFt *v*. FOP labels among these subgroups. Finally, Spearman’s rank correlations tested the correlation between the four self-reported labelling outcomes (NFt awareness and use, FOP label awareness and use).

Statistical analyses were conducted using SAS Studio (SAS Institute). Parameter estimates were reported with 95 % CI. Data were weighted with post-stratification sample weights constructed using population estimates from respective country-based censuses based on age group, gender, region, ethnicity (except in Canada as the national census did not include a simple measure of ethnicity suitable for creating weights) and education (except in Mexico, where the proportion of respondents with lower educational attainment was much smaller than population estimates from census data)^(20)^. All reported estimates are weighted.

## Results

Sample characteristics are presented in Table 2.

**Table 2:** Sample characteristics (*n* 21, 586), International Food Policy Study, 2018*

Characteristic	Australia (*n* 3901)		Canada (*n* 4107)		Mexico (*n* 4012)		UK (*n* 5121)		USA (*n* 4445)	
	%	*n*	%	*n*	%	*n*	%	*n*	%	*n*
Age group										
18–29 years	21·3	831	18·9	777	29·8	1194	19·0	974	20·6	914
30–44 years	26·2	1022	24·7	1014	32·3	1297	24·8	1270	25·1	1115
45–59 years	24·7	963	25·8	1059	28·7	1151	25·9	1327	25·7	1141
≥60 years	27·8	1085	30·6	1257	9·2	370	30·3	1550	28·6	1275
Sex										
Male	48·7	1898	49·4	2028	47·6	1911	47·8	2448	48·2	2141
Female	51·3	2003	50·6	2079	52·4	2101	52·2	2673	51·8	2304
Ethnicity										
Majority	76·1	2969	79·9	3280	78·7	3156	89·1	4563	76·1	3382
Minority	23·9	932	20·1	827	21·3	856	10·9	558	23·9	1063
Education level										
Low	41·6	1622	41·0	1683	19·5	782	47·6	2438	58·2	2585
Medium	32·6	1272	34·1	1400	13·2	531	23·5	1203	10·0	443
High	25·8	1007	24·9	1024	67·3	2699	28·9	1480	31·8	1417
Income adequacy										
Very difficult to make ends meet	8·5	331	8·4	345	12·0	482	6·8	349	9·4	416
Difficult to make ends meet	19·2	750	19·6	804	31·7	1273	18·5	949	20·3	902
Neither easy nor difficult to make ends meet	37·8	1473	36·8	1511	38·9	1559	36·0	1844	33·7	1497
Easy to make ends meet	23·6	921	22·5	927	13·9	557	24·7	1265	21·8	970
Very easy to make ends meet	10·9	426	12·7	520	3·5	141	14·0	714	14·8	660
BMI										
<18·5	3·1	122	3·2	133	2·1	85	2·9	150	3·4	153
18·5–24·9	36·3	1416	33·5	1376	39·6	1588	34·8	1780	31·2	1385
25·0–29·9	26·6	1039	28·8	1183	30·1	1208	27·0	1384	27·6	1226
≥30·0	20·9	815	24·7	1015	15·5	620	17·0	870	27·4	1218
Missing	13·1	509	9·8	400	12·7	511	18·3	937	10·4	463
Food shopping role										
Primary shopper	71·6	2792	72·0	2959	74·9	3005	74·6	3820	73·2	3255
Not primary shopper	6·9	268	5·9	242	5·0	201	4·5	230	6·6	293
Shared equally with others	21·5	841	22·1	906	20·1	806	20·9	1071	20·2	897
Dietary practices										
No specific dietary practices	87·1	3396	90·4	3714	88·2	3539	86·8	4446	88·6	3936
One or more dietary practices (i.e. vegetarian, vegan, pescatarian, religious practices)	12·9	505	9·6	393	11·8	473	13·2	675	11·4	509
	Mean	sd	Mean	sd	Mean	sd	Mean	sd	Mean	sd
Dietary efforts score†	2·7	2·2	2·6	2·1	2·5	2·3	3·0	2·1	2·9	2·3
Health literacy score†	3·23	2·12	3·69	1·97	2·84	1·99	3·19	2·22	3·50	2·12
Nutrition knowledge score†	5·0	1·7	5·1	1·5	4·8	1·5	4·9	1·8	4·6	1·8

* All reported estimates are weighted.

† Mean and sd reported for dietary efforts, health literacy score and nutrition knowledge score.

### Patterns and correlates of nutrition facts table use and awareness

**Fig. 1:**
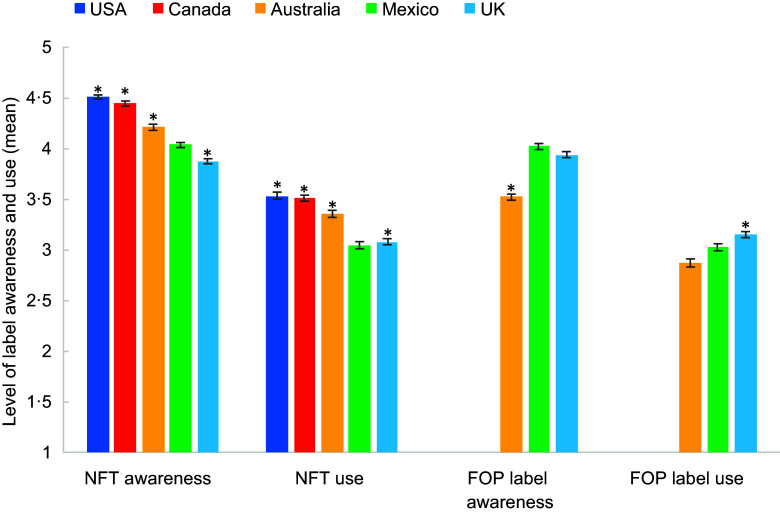
Nutrition facts table and front-of-package label awareness and use by country. Mean levels of awareness and use are shown with 95 % CI. A mean of 1 indicates no awareness/use, and 5 indicates the highest level of self-reported awareness/use. The asterisk denotes significantly different label awareness/use than the reference country (Mexico) at *P* < 0·05. NFT, nutrition facts table; FOP, front-of-package

Figure 1 shows mean NFt use and awareness across countries (categorical responses can be seen in Supplemental Tables 1 and 2). The cross-country data showed that respondents from the USA, Canada and Australia reported significantly higher NFt use than respondents from the UK, and respondents from Mexico reported the lowest use among all countries (see Table 3). Similarly, NFt awareness was highest among respondents from the USA, followed by Canada, Australia, the UK and Mexico. A Spearman rank correlation indicated a moderate correlation between self-reported NFt use and awareness across all countries (*r*_*s*_ = 0·41, *P* < 0·0001).

**Table 3:** Sociodemographic and behavioural correlates of nutrition facts table and front-of-package label use, International Food Policy Study, 2018†

	NFt use (*n* 21 586)			FOP label use (*n* 12 360)		
	*β*	95 % CI	*P*	*β*	95 % CI	*P*
Country						
Australia *v*. Canada	−0·10	−0·15, -0·04	*0·0080	–	–	–
Australia *v*. Mexico	0·36	0·30, 0·42	*<0·0001	−0·02	−0·08, -0·04	0·5339
Australia *v*. UK	0·29	0·24, 0·34	*<0·0001	−0·24	−0·29, -0·19	*<0·0001
Australia *v*. USA	−0·18	−0·24, -0·12	*<0·0001	–	–	–
Canada *v*. Mexico	0·46	0·40, 0·52	*<0·0001	–	–	–
Canada *v*. UK	0·39	0·33, 0·44	*<0·0001	–	–	–
Canada *v*. USA	−0·08	−0·14, -0·02	*0·0053	–	–	–
Mexico *v*. UK	−0·07	−0·13, -0·01	*0·0178	−0·22	−0·28, -0·16	*<0·0001
Mexico *v*. USA	−0·54	−0·60, -0·48	*0·0001	–	–	–
USA *v*. UK	0·47	0·41, 0·52	*<0·0001	–	–	–
Age group						
30–44 *v*. 18–29 years	−0·02	−0·07, 0·03	0·3980	−0·01	−0·07, 0·05	0·7869
30–44 *v*. 45–59 years	0·14	0·09, 0·19	*<0·0001	0·13	0·06, 0·19	*0·0001
30–44 *v*. 60+ years	0·12	0·07, 0·17	*<0·0001	0·20	0·14, 0·27	*<0·0001
45–59 years *v*. 18–29 years	−0·16	−0·22, -0·11	*<0·0001	−0·13	−0·02, -0·06	*0·0002
45–59 years *v*. 60+ years	−0·02	−0·07, 0·03	0·4086	0·08	0·01, 0·14	*0·0229
60+ years *v*. 18–29 years	−0·14	−0·20, -0·08	*<0·0001	−0·21	−0·28, -0·14	*<0·0001
Sex at birth						
Female *v*. male	0·07	0·03, 0·11	*0·0002	0·00	−0·04, 0·05	0·8391
Ethnicity						
Majority *v*. minority	−0·07	−0·12, -0·02	*0·0088	−0·05	−0·12, 0·02	0·1310
Education level						
Medium *v*. low	0·09	0·04, 0·13	*0·0002	0·08	0·02, 0·14	*0·0095
High *v*. low	0·15	0·11, 0·20	*<0·0001	0·21	0·15, 0·37	*<0·0001
High *v*. medium	0·07	0·02, 0·11	*0·0021	0·13	0·07, 0·18	*<0·0001
Income adequacy	0·08	0·06, 0·10	*<0·0001	0·06	0·03, 0·08	*<0·0001
BMI						
<18·5 *v*. 18·5–24·9	−0·02	−0·13, 0·10	0·7483	−0·16	−0·31, -0·02	*0·0270
25–29·9 *v*. ≤18·5	−0·03	−0·14, 0·09	0·6566	0·09	−0·05, 0·24	0·2124
25–29·9 *v*. 18·5–24·9	−0·04	−0·09, 0·00	0·0421	−0·07	−0·12, -0·01	*0·0138
≥30·0 *v*. <18·5	−0·08	−0·20, 0·03	0·1680	0·01	−0·14, 0·17	0·8490
≥30·0 *v*. 18·5–24·9	−0·10	−0·15, -0·05	*<0·0001	−0·15	−0·22, -0·08	*<0·0001
≥30 *v*. 25–29·9	−0·06	−0·11, -0·01	*0·0243	−0·08	−0·15, -0·01	*0·0232
Missing *v*. ≥30	0·08	0·01, 0·15	*0·0184	0·11	0·02, 0·20	*0·0119
Missing *v*. 18·5–24·9	−0·02	−0·08, 0·04	0·5140	−0·04	−0·11, 0·04	0·3388
Missing *v*. <18·5	0·00	−0·12, 0·12	0·9769	0·13	−0·03, 0·28	0·1082
Food shopping role						
Share equally with others *v*. not primary shopper	0·17	0·08, 0·27	*0·0003	0·13	0·01, 0·25	*0·0271
Primary shopper *v*. not primary shopper	0·32	0·23, 0·41	*<0·0001	0·29	0·18, 0·40	*<0·0001
Primary shopper *v*. share equally with others	0·15	0·10, 0·19	*<0·0001	0·16	0·10, 0·22	*<0·0001
Dietary practices						
One or more dietary practices (i.e. vegetarian, vegan, pescatarian and religious practices) *v*. no specific dietary practices	0·39	0·33, 0·45	*<0·0001	0·30	0·23, 0·37	*<0·0001
Dietary efforts score	−0·16	−0·17, -0·15	*<0·0001	−0·13	−0·14, -0·12	*<0·0001
Health literacy status						
Adequate literacy (score 4–6) *v*. high likelihood of limited literacy (score 0–1)	0·07	0·03, 0·12	*0·0027	−0·11	−0·17, -0·06	*<0·0001
Adequate literacy (score 4–6) *v*. possibility of limited literacy (score 2–3)	0·09	0·05,0·14	*<0·0001	−0·07	−0·14, -0·02	*0·0092
Possibility of limited literacy (score 2–3) *v*. high likelihood of limited literacy (score 0–1)	−0·02	−0·07, 0·03	0·4714	−0·04	−0·10, 0·03	0·2675

*β*, parameter estimate; NFt, nutrition facts table; FOP, front-of-package.

* Variables are significant (*P* < 0·05) after *post hoc* adjustment using Benjamini–Hochberg procedure.

† All reported estimates are weighted.

The pattern of sociodemographic characteristics is shown in Table 3. Age, sex, ethnicity, education and BMI were significantly associated with NFt use. Younger respondents reported higher NFt use compared with older respondents. Females reported higher NFt use than males, and respondents from ‘majority’ ethnic groups in their respective countries reported lower NFt use than ‘minority’ ethnic groups. Respondents categorised as having ‘high’ education levels reported higher NFt use than those with ‘medium’ or ‘low’ education, and NFt use was higher with higher income adequacy. Finally, NFt use was lower among respondents with BMI over 30 compared with those with BMI between 18·5–24·9, 25–29·9 and the ‘missing’ category.

Consumers’ dietary behaviours were also associated with NFt label use, as respondents who were primary food shoppers or shared this responsibility equally with others reported higher NFt use than those who were not primary food shoppers in their households. Respondents engaging in vegetarian or other dietary practices, as well as those making efforts to reduce calories, Na, sugars, trans-fats or processed food intake, reported higher use of NFt than those not engaging in specific dietary practices or efforts.

Knowledge-related factors including health literacy status and functional nutrition knowledge were significantly associated with NFt use. Respondents with ‘adequate health literacy’ reported higher NFt use compared with those with a ‘possibility of limited health literacy’ and a ‘high likelihood of limited health literacy’. NFt use was also higher among respondents with higher nutrition knowledge (*β*: 0·07, 95 % CI (0·05, 0·07), *P* < 0·0001).

When comparing NFt use to awareness, a similar pattern of correlates was observed, with the exception of education for which respondents with ‘high’ education reported lower NFt awareness than those with ‘low’ education levels (Table 4). When functional nutrition knowledge was added to this model, NFt awareness was higher among respondents with higher nutrition knowledge scores (*β*: 0·06, 95 % CI (0·05, 0·07), *P* < 0·0001).

**Table 4:** Sociodemographic and behavioural correlates of nutrition facts table awareness, (*n* 21 586), International Food Policy Study, 2018†

	*β*	95 % CI	*P*
Country			
Australia *v*. Canada	−0·19	−0·24, -0·15	*<0·0001
Australia *v*. Mexico	0·14	0·10, 0·19	*<0·0001
Australia *v*. UK	0·34	0·29, 0·38	*<0·0001
Australia *v*. USA	−0·28	−0·32, -0·24	*<0·0001
Canada *v*. Mexico	0·34	0·29, 0·39	*<0·0001
Canada *v*. UK	0·53	0·49, 0·57	*<0·0001
Canada *v*. USA	−0·08	−0·12, -0·04	*<0·0001
Mexico *v*. UK	0·19	0·15, 0·24	*<0·0001
Mexico *v*. USA	−0·42	−0·47, -0·38	*<0·0001
USA *v*. UK	0·61	0·57, 0·66	*<0·0001
Age group			
30–44 years *v*. 18–29 years	−0·08	−0·11, -0·04	*<0·0001
30–44 *v*. 45–59 years	0·03	−0·01, 0·07	0·0967
30–44 *v*. 60+ years	0·03	0·00, 0·07	0·0741
45–59 years *v*. 18–29 years	−0·11	−0·15, -0·07	*<0·0001
45–59 years *v*. 60+ years	0·00	−0·03, 0·04	0·9434
60+ years *v*. 18–29 years	−0·11	−0·15, -0·07	*<0·0001
Sex at birth			
Female *v*. male	0·12	0·09, 0·15	*<0·0001
Ethnicity			
Majority *v*. minority	0·05	0·01, 0·09	*0·0086
Education level			
Medium *v*. low	−0·03	−0·06, 0·01	0·1274
High *v*. low	−0·06	−0·09, -0·03	*0·0002
High *v*. medium	−0·03	−0·06, 0·00	0·0487
Income adequacy	0·03	0·02, 0·05	*<0·0001
BMI			
<18·5 *v*. 18·5–24·9	0·10	0·02, 0·18	*0·0140
25–29·9 *v*. ≤18·5	−0·15	−0·23, -0·07	*0·0003
25–29·9 *v*. 18·5–24·9	−0·05	−0·08, -0·02	*0·0025
≥30·0 *v*. ≤18·5	−0·13	−0·21, -0·04	*0·0023
≥30·0 *v*. 18·5–24·9	−0·03	−0·07, 0·01	0·1220
≥30 *v*. 25–29·9	0·02	−0·01, 0·06	0·2439
Missing *v*. ≥30	−0·07	−0·13, -0·02	*0·0068
Missing *v*. 18·5–24·9	−0·10	−0·15, -0·05	*<0·0001
Missing *v*. <18·5	−0·20	−0·29. -0·11	*<0·0001
Food shopping role			
Share equally with others *v*. not primary shopper	0·03	−0·03, 0·10	0·3113
Primary shopper *v*. not primary shopper	−0·01	−0·07, 0·05	0·6422
Primary shopper *v*. share equally with others	−0·05	−0·08, -0·01	*0·0045
Dietary practices			
One or more dietary practices (i.e. vegetarian, vegan, pescatarian and religious practices) *v*. no specific dietary practices	0·01	−0·03, 0·06	0·5426
Dietary efforts score	−0·06	−0·07, -0·05	*<0·0001
Health literacy status			
Adequate literacy (score 4–6) *v*. high likelihood of limited literacy (score 0–1)	0·49	0·46, 0·53	*<0·0001
Adequate literacy (score 4–6) *v*. possibility of limited literacy (score 2–3)	0·21	0·18, 0·24	*<0·0001
Possibility of limited literacy (score 2–3) *v*. high likelihood of limited literacy (score 0–1)	0·28	0·24, 0·32	*<0·0001

*β*, parameter estimate; NFt, nutrition facts table.

* Variables are significant (*P* < 0·05) after *post hoc* adjustment using Benjamini–Hochberg procedure.

† All reported estimates are weighted.

There were differential patterns across countries for NFt use based on age, sex, ethnicity, education level, income adequacy, health literacy and dietary efforts (see online Supplemental Table 3). Women in Mexico reported lower NFt use than UK women; however, Mexican respondents with ‘high’ education and income adequacy reported higher NFt use compared with respondents in the UK with similar education and income adequacy. Australian respondents with ‘adequate health literacy’ reported higher NFt use than ‘adequate health literacy’ respondents in the UK. For NFt awareness, Canadian respondents with ‘high’ education reported lower NFT awareness than those with similar education in the UK. Mexican respondents with ‘adequate literacy’ and a ‘possibility of limited literacy’ reported lower NFT awareness than the corresponding health literacy groups in the UK.

### Patterns of front-of-package labelling and correlates

Cross-country data in the three countries with FOP labelling policies found that respondents from Mexico reported the highest awareness of FOP labels (mean 4·0), followed by the UK (mean 3·9) and Australia (mean 3·5) (Fig. 1). In addition, respondents in the UK reported the highest FOP label use (mean 3·2) and Australia the lowest (mean 2·9). FOP label use and awareness were moderately correlated (*r*_*s*_ = 0·39, *P* < 0·0001). Correlates of FOP label use were similar to NFt use, with a few exceptions (Table 3). A review of sociodemographic and other correlates found that sex and ethnicity were not significantly associated with FOP label use, and respondents with ‘adequate literacy’ reported lower FOP label use compared with those with a ‘high likelihood of limited literacy’. Nutrition knowledge score was not significantly associated with FOP label use (*β*: 0·01, 95 % CI (0·00, 0·02), *P* = 0·1978).

### Use and awareness of nutrition facts table *v.* front-of-package labels

Significant differences were found between NFt and FOP label use and awareness within countries with both label types. In Australia, respondents reported higher use and awareness of NFt compared with voluntary FOP Health Star Ratings. As shown in Table 4, respondents aged ≥60 years were more likely to be aware of and use NFt than Health Star Ratings compared with 18–29-year-olds. Female respondents and those with higher income adequacy were also more likely to use NFt than Health Star Ratings. Respondents with ‘adequate literacy’ were more likely to report higher NFt than FOP label use and awareness compared with those with a ‘high likelihood of limited literacy’. Respondents with higher nutrition knowledge were more likely to use and be aware of NFt than FOP labels. Specific dietary practices or efforts to consume less of specific nutrients (i.e. sugar, Na and trans fat) were associated with higher NFt than FOP label use, and primary food shoppers were less likely to be aware of NFt than FOP labels compared with respondents who were not primary food shoppers in their households.

In the UK, respondents reported lower NFt use and awareness compared with the voluntary FOP Traffic Light labels. Older age groups (60+, 45–59 and 30–44 years compared with 18–29 years) were more likely to be aware of or use NFt compared with FOP Traffic Lights. Respondents who identified as belonging to the ‘majority’ ethnic group in the UK were more likely to report higher FOP label than NFt use and awareness compared with those from ‘minority’ ethnic groups. Respondents with ‘high’ education levels were significantly more likely to be aware of NFt than FOP labels compared with respondents with ‘medium’ education levels. Similarly, respondents with ‘adequate literacy’ were more likely to report higher use and awareness of NFt than FOP labels compared with respondents with a ‘high likelihood of limited literacy’. Respondents engaging in efforts to consume less Na, sugar, trans-fat, processed food or calories were more likely to use FOP labels than NFt.

In Mexico, respondents reported higher NFt use and awareness compared with Guideline Daily Amount labels. Older age groups and females were more likely to report higher NFt than FOP (Guideline Daily Amount) label awareness compared with 18–29-year-olds and males, respectively. Respondents who reported higher nutrition knowledge and those with ‘adequate literacy’ were more likely to report higher FOP label than NFt awareness compared with those with lower nutrition knowledge scores or a ‘high likelihood of limited literacy’, respectively. Dietary efforts to consume less of specific nutrients were also associated with higher FOP label than NFt use. There were no significant differences between NFt and FOP label use among the subgroups tested in Mexico. Interactions between country and BMI were not significant for NFt awareness or use in Australia, the UK or Mexico.

## Discussion

Multi-country, population-level studies are important for ascertaining which labelling policies are most effective. Country-specific differences in label awareness and use provide insights into which labels have the greatest reach among consumers from varied subgroups. Evidence has shown greater uptake for mandatory labelling policies^(10)^, consistent with findings from this study demonstrating significantly higher NFt use compared with voluntary FOP labelling systems (with the exception of Traffic Lights in the UK), and higher awareness of the mandatory FOP Guideline Daily Amount label in Mexico compared with voluntary FOP labelling systems. NFt have been a long-standing policy in all five countries^(28–32)^; hence, high levels of use and awareness were not surprising. Significantly higher levels of NFt awareness and use observed in this study were in the USA. As the first country to enact nutrition labelling policies, as well as a greater reliance on processed, packaged foods, higher levels of NFt awareness among Americans may be attributed, in part, to these factors^(5)^.

Among countries with FOP labelling systems, Guideline Daily Amount labels in Mexico had the lowest level of reported use, despite high levels of awareness. Mexico is the only country in this study with a mandatory FOP label, so greater awareness of Guideline Daily Amounts may have stemmed from relatively higher exposure to this label on the FOP compared with voluntary FOP labels^(10)^. The voluntary nature of FOP labels in Australia and the UK may account for lower levels of awareness and use compared with mandatory NFt and may have resulted in lower FOP label exposure as Traffic Light labels and Health Star Ratings are estimated to appear on approximately 8 % and 30 % of food products, respectively^(13,33)^. Lower awareness of voluntary labels supports consideration of mandatory FOP labelling policies and also reiterates the importance of closely monitoring policy implementation.

Existing evidence also highlights that not all FOP labels are equal. The finding that self-reported use of the mandatory Guideline Daily Amount label in Mexico was significantly lower than voluntary FOP label use in Australia and the UK is consistent with literature documenting consumers’ difficulty understanding these labels^(11,34–37)^. Indeed, the Mexican government is replacing Guideline Daily Amounts with mandatory FOP ‘high-in’ labels similar to Chile^(38)^, as emerging evidence demonstrates ease of use and greater understanding of this simple, interpretative label format^(39,40)^. While the present study did not examine the impact of mandatory *v*. voluntary nutrition labels on product reformulation, evidence has shown that labels have the potential to incentivise healthier reformulation of packaged foods that exceed thresholds for nutrients of public health concern^(8,9,41)^. There is also encouraging evidence of positive impacts of mandatory labelling on consumers’ food purchasing, with one longitudinal study in Chile finding increased healthy food and decreased sugar-sweetened beverage purchases after the implementation of FOP warning labels^(42)^.

Differences in nutrition label use and awareness based on consumer knowledge-related factors (i.e. health literacy status, functional nutrition knowledge) and sociodemographic characteristics have important policy implications, particularly regarding the design of accessible nutrition labels. Consistent with the literature^(15,16)^, this study found that respondents with higher education, health literacy status and functional nutrition knowledge reported higher NFt use and awareness, likely reflecting better numeracy skills and ability to understand label information^(24,34)^. Evidence has shown that consumers with lower literacy or nutrition knowledge may be at a disadvantage for applying nutrition information from labels, which could limit their ability to make healthier purchasing decisions^(43–45)^. In response, FOP labels were designed to make nutrition information simpler to interpret; thus, higher self-reported FOP label use among those with lower health literacy status suggests greater accessibility of interpretative FOP label information compared with NFt.

Despite different rates of usage, there were many similarities in the correlates of NFt and FOP label use, with generally higher use among primary food shoppers, respondents with specific dietary practices or diet modification efforts, respondents with BMI under 30, and females compared with males. Research has shown that consumers following specific dietary practices or with diet-related goals have increased motivation to seek out nutrition information, which likely drives higher label use^(18,19,25)^. While primary food shoppers may not necessarily be making specific dietary efforts, they may be making food choices for others in their household (i.e. children), potentially motivating greater use of labels than those who are not primary food shoppers^(25)^. Moreover, studies have found women to be more health conscious than men, leading to greater use of nutrition information^(12,46)^. In order for the general public to take an interest in nutrition labelling policy, health promotion campaigns which aid consumers in identifying how good nutrition fits in the broader context of their health may encourage label use. Pairing complementary initiatives together (i.e. nutrition labelling and school-based curricula) can increase awareness and accessibility of nutrition labelling information beyond groups that have a vested interest in nutrition information.

Several sociodemographic characteristics were significantly associated with nutrition label use, with generally lower use among older age groups and those with lower income adequacy. One potential explanation for lower label use among older individuals may be brand or product familiarity^(12)^. Studies on product health claims have shown that consumers who are familiar with a product are less likely to read labels or claims^(12,47)^; hence, NFt and FOP labels may not be used by habitual consumers unless they are considering a new brand or product^(48)^. Households with low incomes report prioritising accessibility and affordability when making food purchasing and consumption decisions^(44,49)^. As a result, these consumers may report using nutrition label information less often due to other priorities aside from nutrition quality. Understanding the sociodemographic characteristics that drive nutrition label use is critical for policymakers to consider, particularly in shaping the broader nutrition environment. For example, consumers prioritising factors such as affordability over product healthiness reiterates the need for a system-wide approach to improving the food system. Prompted by mandatory nutrition labelling^(13,41)^, research has shown promising improvements in healthier product reformulation, a system-level strategy which is necessary given the prominence of ultra-processed foods^(1,3)^.

There are very few studies exploring label use differences by ethnicity^(50)^, and this study found higher use of NFt among ‘minority’ respondents. The dearth of literature exploring disparities in the reach of nutrition labelling policy limits our ability to unpack why some ethnic groups use labels more or less. For example, in a study of menu label use, Feng & Fox (2018) found that Black and Hispanic groups used labels more than their White counterparts at sit-down restaurants^(50)^, whereas another study in New Zealand found lower use of NFt labels among minority ethnic groups^(42)^. More research is required to better understand what other factors may be driving NFt use, and whether immigrant status or cultural food preferences may play a role in product familiarity and label use.

This study compared label awareness and use between five countries, with a large sample that enabled consideration of a range of covariates. A limitation is the non-probability-based sampling strategy, which does not enable the generation of nationally representative population estimates. Although analyses included post-stratification weights to make the sample more similar to the age, sex, region and ethnicity distributions in each country, the Mexico sample had higher educational attainment than in the Mexican population, while self-reported BMI was lower than national estimates in each of the five countries^(20)^. Moreover, the primary outcomes (NFt/FOP label awareness/use) and several other correlates (nutrition knowledge score, BMI) are subject to social desirability bias given the use of self-reported measures. In addition, the Newest Vital Sign has been tested across a variety of age and ethnic groups in different countries but has not yet been validated for online, self-administration^(17,24)^; however, that pattern of results we found provide evidence of its construct validity across countries. Lastly, the cross-sectional study design limits possible conclusions about the direction of variable relationships such as label use and nutrition knowledge.

## Conclusions

The study findings are relevant as an increasing number of countries adopt voluntary or mandatory FOP labelling policies. Lower use of Guideline Daily Amount labels compared with voluntary FOP labelling systems provides further support for Mexico’s decision to switch to mandatory FOP ‘high-in’ symbols. Sociodemographic and other subgroup differences in label use are important as they indicate the reach of various labelling policies, which can potentially translate to dietary choices. Considering the varied use and awareness of nutrition labels among consumers in each country, accessible nutrition labelling policies (i.e. use of FOP labels) as well as broader nutrition promotion initiatives which address the diversity of consumers (i.e. label promotion campaigns in different languages) can aid efforts to improve diet quality. Future research should investigate the implications of labelling policies on consumers’ eating patterns over time, including countries with mandatory FOP labelling policies, as well the impact of nutrition labelling policies on food reformulation.

## Supplementary material

For supplementary material accompanying this paper visit https://doi.org/10.1017/S1368980022002257. click here to view supplementary material
