# Supplementary material for: A five-country study of front- and back-of-package nutrition label awareness and use: patterns and correlates from the 2018 International Food Policy Study
Source: Public Health Nutr. 2022 Oct 26;26(1):275–86. doi: 10.1017/S1368980022002257 (PMC11077451; doi:10.1017/S1368980022002257)
Supplement: Supplementary file 1 [file S1368980022002257sup001.docx]

**Supplementary Table 1: Nutrition label use across countries^1^**

| **Response** | **NFt use, % (n)** | | | | | | **FOP label use, % (n)** | | | |
| --- | --- | --- | --- | --- | --- | --- | --- | --- | --- | --- |
|  | ***All countries*** | ***Australia*** | ***Canada*** | ***Mexico*** | ***UK*** | ***US*** | ***All countries*** | ***Australia*** | ***Mexico*** | ***UK*** |
| 1 – Never | 8.7 (1877) | 7.5  (293) | 6.9  (285) | 11.4  (457) | 11.2  (575) | 6.0  (267) | 10.9 (1342) | 12.0 (410) | 11.2  (441) | 9.9  (491) |
| 2 – Rarely | 16.3 (3522) | 15.3  (597) | 12.4  (511) | 20.8  (833) | 19.5  (1000) | 13.1  (581) | 19.9 (2466) | 20.8 (713) | 21.5  (848) | 18.1 (904) |
| 3 –Sometimes | 31.6 (6817) | 31.0  (1209) | 29.2  (1201) | 30.8  (1237) | 35.9  (1836) | 30.0  (1335) | 35.7 (4412) | 40.2 (1378) | 31.8  (1256) | 35.7  (1778) |
| 4 – Often | 27.1 (5852) | 27.8  (1085) | 31.1  (1277) | 26.5  (1062) | 22.6  (1158) | 28.6  (1270) | 23.6 (2912) | 20.0 (687) | 24.9  (981) | 25.0  (1244) |
| 5- All the time | 16.3 (3517) | 18.4  (717) | 20.3  (834) | 10.6  (423) | 10.7  (552) | 22.3  (992) | 9.9 (1228) | 7.1 (242) | 10.7  (421) | 11.3  (565) |

^1^NFt – Nutrition Facts table. FOP – front-of-package. UK – United Kingdom. US – United States. Label use was measured by asking, “How often do you use this type of food label when deciding to buy a food product?” Sample size for NFt use is 21,586 and 12,360 for FOP label use.

**Supplementary Table 2: Nutrition label awareness across countries^1^**

| **Response** | **NFt awareness, % (n)** | | | | | | **FOP label awareness, % (n)** | | | |
| --- | --- | --- | --- | --- | --- | --- | --- | --- | --- | --- |
|  | ***All countries*** | ***Australia*** | ***Canada*** | ***Mexico*** | ***UK*** | ***US*** | ***All countries*** | ***Australia*** | ***Mexico*** | ***UK*** |
| 1 – Never | 0.0  (0) | 0  (0) | 0  (0) | 0  (0) | 0  (0) | 0  (0) | 0.0  (2) | 0.0 (0) | 0  (0) | 0.0 (2) |
| 2 – Rarely | 5.0 (1085) | 4.5  (176) | 3.5  (143) | 5.5  (222) | 7.8  (399) | 3.3  (146) | 8.0  (985) | 12 (412) | 7.0  (277) | 6.0  (297) |
| 3 – Sometimes | 17.2 (3716) | 17.0  (665) | 9.5  (389) | 19.5  (783) | 28.0  (1434) | 10.0  (446) | 27.8 (3441) | 37.8 (1297) | 21.7  (855) | 25.9  (1288) |
| 4 – Often | 31.4 (6771) | 32.5  (1268) | 25.4  (1044) | 42.5  (1707) | 35.8  (1834) | 20.7  (919) | 36.6 (4528) | 33.9 (1163) | 38.1  (1506) | 37.3  (1859) |
| 5- All the time | 46.4 (10014) | 45.9  (1792) | 61.7  (2533) | 32.4  (1300) | 28.4  (1455) | 66.0  (2934) | 27.5 (3403) | 16.3  (558) | 33.2 (1310) | 30.8  (1536) |

^1^NFt – Nutrition Facts table. FOP – front-of-package. UK – United Kingdom. US – United States. Label awareness was measured by asking, “How often have you seen this type of food label in packages or in stores?” Sample size for NFt awareness is 21,586 and 12,360 for FOP label awareness.

**Supplementary Table 3:** **Two-way interactions for five-country regression models on NFt awareness and use (n=21,586), International Food Policy Study, 2018^1^**

|  | **NFt awareness**  **β (CI), p-value** | **NFt use**  **β (CI), p-value** |
| --- | --- | --- |
| **Country x Age Group**, p=<.0001 (awareness and use) | | |
| Australia*30-44 years | -0.11 (-0.24, 0.02),  p = 0.1068 | -0.15 (-0.32, 0.01),  p = 0.0705 |
| Canada*30-44 years | -0.14 (-0.27, -0.01),  p = 0.0260* | -0.16 (-0.33, 0.02),  p = 0.0808 |
| Mexico*30-44 years | -0.15 (-0.27, -0.03), p = 0.0107* | 0.00 (-0.15, 0.15),  p = 0.9747 |
| UK*30-44 years | Ref | |
| Mexico*45-59 years | -0.06 (-0.18, 0.07),  p = 0.4036 | 0.39 (0.23, 0.56),  p <.0001* |
| UK*45-59 years | Ref | |
| Canada*60+ years | 0.18 (0.05, 0.31),  p = 0.0059* | 0.31 (0.09, 0.53),  p = 0.0050* |
| Australia*60+ years | 0.22 (0.09, 0.35),  p = 0.0010* | 0.11 (-0.06, 0.29),  p = 0.1893 |
| Mexico*60+ years | 0.10 (-0.07, 0.25),  p = 0.2640 | 0.31 (0.09, 0.53), p=0.0050* |
| US*60+ years | 0.26 (0.13, 0.37)  p <.0001* | 0.17 (0.00, 0.35),  p = 0.0480 |
| UK*60+ years | Ref | |
|  | | |
| **Country x Sex**, p=0.0298 (awareness) and 0.0330 (use) | | |
| Mexico*Female | -0.13 (-0.21, -0.04), p = 0.0040* | -0.12 (-0.23, -0.01), p = 0.0329* |
| US*Female | -0.08 (-0.16, -0.00), p = 0.0611 | 0.07 (-0.04, 0.18),  p = 0.2094 |
| UK*Female | Ref | |
|  | | |
| **Country x Education Level**, p = 0.1135 (awareness) and 0.0151 (use) | | |
| Canada*High education | -0.14 (-0.23, -0.04), p=0.0052* | 0.02 (-0.11, 0.16),  p = 0.7162 |
| Mexico*High education | 0.03 (-0.08, 0.13),  p = 0.6231 | 0.24 (0.09, 0.28), p = 0.0011* |
| UK*High education | Ref | |
|  | | |
| **Country x Income Adequacy**, p= 0.0195 (awareness) and 0.0005 (use) | | |
| Mexico*Income adequacy | 0.00 (-0.04, 0.04),  p = 0.9361 | 0.07 (0.02, 0.13), p=0.0095* |
| UK*Income adequacy | Ref | |
|  | | |
| **Country x Ethnicity**, p= 0.0205 (awareness) and 0.6785 (use) | | |
| Canada*Majority | 0.17 (0.03, 0.30),  p = 0.0160* | 0.00 (-0.18, 0.18), p = 0.9894 |
| Mexico*Majority | 0.15 (0.00, 0.30),  p = 0.0513 | -0.10 (-0.29, 0.08),  p = 0.2879 |
| US*Majority | 0.13 (0.00, 0.28),  p = 0.0515 | 0.00 (-0.17, 0.17),  p = 0.9857 |
| UK*Majority | Ref | |
|  | | |
| **Country x Health Literacy Status**, p=<.0001 (awareness) and 0.0027 (use) | | |
| Australia*Adequate literacy | -0.02 (-0.13, 0.08),  p = 0.6552 | 0.17 (0.04, 0.31),  p = 0.0099* |
| US*Adequate literacy | 0.07 (-0.04, 0.18),  p = 0.2386 | -0.11 (-0.25, 0.04),  p = 0.1415 |
| Mexico*Adequate literacy | -0.31 (-0.41, -0.19), p<.0001* | -0.07 (-0.21, 0.07),  p = 0.3052 |
| UK*Adequate literacy | Ref | |
| Mexico*Possibility of limited literacy | -0.21 (-0.33, -0.08), p = 0.0013* | -0.04 (-0.20, 0.11),  p = 0.5832 |
| UK*Possibility of limited literacy | Ref | |
|  | | |
| **Country * Dietary Efforts Score**, p <.0001 (awareness and use) | | |
| Canada*Dietary efforts | 0.00 (-0.02, 0.02),  p = 0.8556 | -0.03 (-0.06, 0.00)  p = 0.0277* |
| Mexico*Dietary efforts | 0.06 (0.04, 0.08), p<.0001* | 0.06 (0.03, 0.09), p<.0001* |
| US*Dietary efforts | 0.02 (0.00, 0.04), p=0.0221* | 0.00 (-0.02, 0.03),  p = 0.6408 |
| UK*Dietary efforts | Ref | |

^1^NFt – Nutrition Facts table. β - parameter estimate. CI -Confidence Interval. US – United States. UK – United Kingdom. Ref – reference category. Regression model adjusted for sociodemographic (age, sex, country), socioeconomic (education, income adequacy), dietary behaviors (food shopping role, dietary practices, dietary efforts), body mass index, and health literacy. *Variables are significant (p<0.05). The overall effect of country interactions with body mass index, food shopping role and dietary practices were not significant for NFt use or awareness. All reported estimates are weighted.
